# Supplementary material for: Construction and Validation of the Positive Mental Health Literacy Assessment Scale in Adults
Source: Int J Environ Res Public Health. 2023 Jul 18;20(14):6391. doi: 10.3390/ijerph20146391 (PMC10379995; doi:10.3390/ijerph20146391)
Supplement: Supplementary file 1 [file ijerph-20-06391-s001.zip › ijerph-2358252-supplementary.pdf]

Table S1 Escala de Avaliação da Literacia em Saúde Mental Positiva em Adultos

[Positive Mental Health Literacy Assessment Scale in Adults]

	Nada importante [Not at all important]	Pouco importante [Not very important]	Mais ou menos importante [Somewhat important]	Importante [Important]	Muitíssimo importante [Very important]
1 Ter capacidade para lidar com problemas inesperados. [Be able to deal with unexpected situations]					
2 Ter capacidade para lidar com as exigências do dia-a-dia. [Be able to deal with everyday demands]					
3 Ter atividades de diversão. [Have leisure activities]					
4 Dedicar tempo semanal a uma atividade de lazer. [Spend time in a weekly leisure activity]					
5 Ter relações de cordialidade com os vizinhos. [Have cordial relations with neighbours]					
6 Participar em atividades da comunidade (desportivas, festividades religiosas, eventos festivos, …) [Participate in community activities (sports, religious festivities, festive occasions]					
7 Conhecer a comunidade, as pessoas e as instituições existentes da sua zona de residência. [To get to know the community, people, and institutions in the area of residence]					
8 Manter práticas culturais ao longo do ano (dias festivos, religiosos, …). [Maintain cultural practices throughout the year (festive, religious days, …]					
9 Ter capacidade para gerir situações stressantes ou angustiantes. [Be able to manage stressful situations]					
10 Acreditar em si mesmo. [Believe in oneself]					
11 Sentir-se apoiado pelos profissionais de saúde. [Feel support from health professionals]					
12 Ser capaz de gerir a sua saúde. [Be able to manage one's health]					
13 Ter apoio social quando necessita. [Having social support when needed]					
14 Ter capacidade para compreender as informações relacionadas com a saúde. [Be able to understand health-related information]					
15 Ter capacidade para avaliar as informações relacionadas com a saúde que lhe são úteis. [Be able to evaluate health-related information for self-purpose]					
16 Ter capacidade para encontrar a informação adequada sobre a saúde. [Be able to find appropriate health-related information]					
17 Ter atitudes de aceitação do outro. [Be accepting of others]					
18 Praticar um estilo de vida saudável. [Have a healthy lifestyle]					
19 Manter um relacionamento significativo. [Maintain a meaningful relationship]					
20 Definir limites para as próprias ações. [Set boundaries for self-action]					

Table S1 Escala de Avaliação da Literacia em Saúde Mental Positiva em Adultos

[Positive Mental Health Literacy Assessment Scale in Adults]

21 Praticar técnicas de meditação. [Practice mediation techniques]					
22 Uso de técnica de relaxamento. [Use of relaxation techniques]					
23 Falar com os pais, filhos ou outra pessoa significativa. [Talk to parents, children or significant other]					
24 Estar com as pessoas que ama. [Be with significant persons]					
25 Ter uma atitude positiva. [Have a positive attitude]					
26 Ter relações interpessoais com qualidade. [Have quality interpersonal relationships]					
27 Realizar atividades de promoção da saúde mental. [Perform activities of mental health promotion]					
28 Estar envolvido na tomada de decisões sobre cuidados de saúde. [Be involved in health care decision-making]					
29 Tomar decisões sobre as atividades de vida quotidiana. [Decide on the daily life activities]					
30 Procurar informações úteis. [Seek useful information]					
31 Participar na tomada de decisões da vida. [Participate in life decision-making]					
32 Sentir que pertence a um grupo ou comunidade. [Feeling of belonging to a group or community]					
